# Breast density in polycystic ovarian syndrome patients: A case-control study

**DOI:** 10.18502/ijrm.v17i8.4823

**Published:** 2019-09-03

**Authors:** Bita Eslami, Sadaf Alipour, Reihaneh Hosseini, Bentolhoda Fattah, Ashraf Moini

**Affiliations:** ^1^Breast Disease Research Center (BDRC), Tehran University of Medical Sciences Tehran Iran.; ^2^Department of Surgery, Arash Women's Hospital, Tehran University of Medical Sciences Tehran Iran.; ^3^Department of Gynecology and Obstetrics, Arash Women's Hospital, Tehran University of Medical Sciences Tehran Iran.; ^4^Department of Endocrinology and Female Infertility at Reproductive Biomedicine research center, Royan Institute for Reproductive Biomedicine, ACECR Tehran Iran.

**Keywords:** Breast density, Mammographic, Polycystic ovarian syndrome, Breast cancer

## Abstract

**Background:**

Epidemiological studies suggested a positive relationship between breast density and risk of breast cancer. One of the common hormonal disorders in women's reproductive age is polycystic ovarian syndrome (PCOS) and the results from the studies about the risk of breast cancer among PCOS patients are equivocal.

**Objective:**

The objective was to evaluate the breast density in PCOS patients compared with the control group.

**Materials and Methods:**

In this case-control study, the PCOS patients who were older than 40 years and were referred to infertility or gynecology outpatient clinic of Arash women's hospital between 2015 and 2017 were selected as the case group. Control group was selected from healthy women who attended the same hospital and were older than 40 years. By digital mammography, breast density was classified according to the Breast Imaging Reporting and Data System (BIRADS) of the American College of Radiology and it was graded by one expert radiologist.

**Results:**

Final analysis in 68 cases and controls showed statistically significant differences between breast densities in PCOS patients compared to the control (p░=░0.03), and when the analysis was conducted by considering the category of age, the control group who were younger than 45 years had higher breast density compared with PCOS patient. Multivariate logistic regression analyses manifested a statistically significant adverse association between body mass index (OR░=░0.87, 95% CI: 0.79–0.95), vitamin D intake (OR░=░0.35, 95% CI: 0.16–0.81), and breast density.

**Conclusion:**

Our data suggested that the PCOS patients had lower breast density compared with normal population. However, in multivariate analysis, considering other confounders, this association was not confirmed.

## 1 Introduction

Breast density is a measure used to describe women's mammogram by the amount of fibrous and glandular tissue. The issue of breast density and its relation to breast cancer has been a hot topic over the last several years. A meta-analysis study in 2006 manifested the strong association between breast density and breast cancer (Relative risk: 4.64, 95%CI: 3.63–5.91) for the high density compared with the low dense category (1). A recent biological study in 2018 revealed in dense breast tissue the transition of breast epithelial cells from normal to malignant cells is more frequent compared with the non-dense tissue (2). Therefore, the amount of mammographic density is one of the risk factors for breast cancer (3). Researchers investigated the effects of different variables such as age, reproductive and hormonal factors, body size, and anthropometric characteristics on breast density (4–7). Epidemiological studies reported the relationship between breast density and hormonal factors associated with infertility such as anovulation or other forms of luteal deficiency (6, 8). One of the common hormonal disorders in women's reproductive age is polycystic ovarian syndrome (PCOS), and using different criteria, the prevalence in different geographic regions of Iran was reported to be 7.1–14.6% (9). The results from the studies about the risk of breast cancer among PCOS patients are equivocal (10–13). Recent finding from a systematic review and meta-analysis in 2014 did not manifest the relationship between ovarian and breast cancer with PCOS in all ages (14). However, a nationwide population-based retrospective cohort study concluded the possible increasing the risk of breast cancer in PCOS patients (15). A study in Iran was conducted to evaluate the prevalence of PCOS in premenopausal breast cancer patients and they found no relation between PCOS and breast cancer (16). Since the prevalence of the risk factors of cancer is high in Iran, and the trend is growing up (17) and breast cancer patients with advanced stages of disease are relatively younger (about 10░yr) than their western counterpart (18), assessing the risk of breast cancer in probable high-risk population is necessary in order to prevention and early detection of this disease.

Therefore, the objective of this study was to compare the breast density as a known risk factor for breast cancer in patients with PCOS in comparison with a control group in Iranian women population.

## 2 Materials and Methods

This case-control study is conducted on 136 women who attended Arash women's hospital in Tehran, Iran, during 2015–2017. Case group was selected from women referred to infertility or gynecology outpatient clinic and PCOS was confirmed in them.

PCOS was diagnosed according to the Rotterdam criteria (at least two of the following criteria): oligo/amenorrhea, clinical or biochemical hyperandrogenism, and polycystic ovaries (PCO) on ultrasonography (10 or more peripheral follicular cysts at most 8░mm in diameter in one plane along with increased central ovarian stroma) (19). Control group was selected from women who attended the same hospital, and sonographic and clinical evaluation revealed that they had normal ovaries and PCOS was not diagnosed in them.

The exclusion criteria for both the groups were: Cushing syndrome, late-onset adrenal hyperplasia, androgen-producing neoplasm, hyperprolactinemia, previous history of breast cancer or breast surgery, renal failure, and usage of estrogen, progesterone, and androgen. The age of all participants was more than 40░yr and they were in premenopausal status. Breast screening was performed by digital mammography in two standard mediolateral-oblique and craniocaudal views for all normal-risk patients 40░yr of age or more as a routine in Arash woman's hospital. Breast Imaging Reporting and Data System (BIRADS) that was established by the American College of Radiology (ACR) was used to classify breast density. Four BIRADS categories for breast density was identified by an expert radiologist. BIRADS 1 and 2 were considered as low density and 3 and 4 as high density. The trained physician gathered individual information about age, age of menarche, weight, height, waist and hip circumference, menstrual pattern, parity, previous history of abortion, hormone therapy, vitamin D and calcium consumption, daily sun exposure (≤ 30 and > 30░min), and family history of breast surgery by in-person interviews. Age was categorized into two groups (< 45 and ≥ 45░yr). Body mass index (BMI) is defined as weight divided by height squared (kg/m^2^), and it was classified according to the WHO classification (< 18.5, 18.5–24.99, 25–29.99, ≥ 30). The waist and hip circumferences of each participant were used to construct a waist-hip ratio (WHR cm/cm). Both Active and passive smoking was considered as smoking status. Having more than 10░minutes of any activity per day that led to increased heartbeat and breathing was defined as physical activity.

### 2.1 Sample size

Based on the results of previous study in our population (20), the prevalence of high density in the control group was about 40%. In order to detect 25% difference in the prevalence of high-density breast between two groups, we calculated that 70 people will be required in each group with the power of 80% and α░=░0.05 by using the Epi Info website (www.cdc.gov/epiinfo/).

### 2.2 Ethical consideration

The present study was approved by the ethics committee of Tehran University of Medical Sciences (code: IR.TUMS.REC.1394.254) and an informed consent was obtained from all individual participants included in the study.

### 2.3 Statistical analysis

Statistical Package for the Social Sciences software (SPSS, version 18.0, Chicago, IL, USA) was used for statistical analysis. Categorical and continuous variables are expressed as number (%) and mean░±░standard deviation, respectively. The differences between variables were tested with Pearson chi-square tests and independent *t*-test. Multivariate logistic regression analysis was used to examine the association between breast density (low/high) as the dependent variable and other independent variables. In the multivariate model, variables were entered to the model based on our previous knowledge and p░<░0.2, whereas p░=░0.1 was the threshold for a variable to stay in the model. Results are presented as odds ratio (OR) with 95% confidence intervals (CI). P-value less than 0.05 was considered as statistically significant and all tests were two-sided.

## 3 Results

Final analysis was performed with 68 samples in each group. Table I shows the total characteristics of the study sample. PCOS patients had a significantly higher BMI, WHR, and hormone therapy as expected (p░<░0.05). Meanwhile, PCOS patients were in more exposure to cigarette smoke (active or passive), had more physical activity and calcium intake than the other group.

**Table I:** Total characteristics of case (PCOS) and control groups

Variables	PCOS (n░=░68)	Control (n░=░68)	P-value
Age (yrs)^*^	45.24░±░4.07	45.73░±░4.22	0.50
BMI (kg/m^2^) ^*^	29.42░±░6.67	27.58░±░4.41	0.06
WHR^*^	0.88░±░0.09	0.83░±░0.07	0.001
Parity (n)^*^	2.42░±░1.09	2.28░±░1.02	0.48
Age at first pregnancy (yr)^*^	23.49░±░6.66	21.94░±░5.16	0.18
Age of menarche (yr)^*^	13.71░±░1.80	13.63░±░2.45	0.84
The family history of breast surgery^**^	17 (25)	10 (14.7)	0.20
Exposure to cigarette smoke^**^	20 (29.4)	10 (14.7)	0.06
Physical activity^**^	53 (77.9)	29 (42.6)	< 0.001
History of breastfeeding^**^	52 (76.5)	54 (79.4)	0.84
Daily sun exposure more than 30░min^**^	4 (5.9)	15 (22.1)	0.01
History of abortion^**^	22 (32.4)	16 (23.5)	0.34
Vitamin D intake^**^	24 (35.3)	18 (26.5)	0.35
Calcium intake^**^	64 (94.1)	51 (75)	0.004
Hormone therapy^**^	37 (54.4)	4 (5.9)	< 0.001
ACR breast density^**^ 1 2 3 4	16 (23.5) 22 (32.4) 21 (30.9) 9 (13.2)	4 (5.9) 26 (38.2) 30 (44.1) 8 (11.8)	0.03

* Data expressed as mean░±░SD;

** Data expressed as number (%)

P-value refers to *t*-test in comparison of continues variables and Chi-square test was conducted between categorical variables

PCOS: Polycystic ovarian syndrome; BMI: Body mass index; WHR: Waist-hip ratio

The results revealed statistically significant differences between breast densities in PCOS patients compared to the control (p░=░0.03). When the analysis was conducted by considering the category of age the control group who were younger than 45░yr old had higher breast density compared with PCOS patient (Table II). Whereas, no association was found between the two groups with ≥ 45░yr old. Table III shows the comparison of all variables between low- and high-density group. Overall, statistically significant difference was observed in BMI, vitamin D intake, and family history of breast surgery between low- and high-density group. The results of multivariate logistic regression analysis considering the following covariates: group (PCOS/control), age (yr), BMI (kg/m^2^), WHR, vitamin D intake (no/yes), physical activity, and positive family history of breast surgery (no/yes) were manifested in Table IV. The adjusted OR illustrated a statistically significant adverse association between BMI (OR░=░0.87, 95% CI: 0.79–0.95) and vitamin D intake (OR░=░0.35, 95% CI: 0.16–0.81) with breast density. In addition, the family history of breast surgery (OR░=░2.54, 95% CI: 0.96–6.67) had a positive association with breast density with a borderline p░=░0.06.

**Table II:** Comparison of breast density in two groups considering age category

Age category	Group	Low density	High density	P-value
< 45░yr	PCOS	26 (63.4)	15 (36.6)	0.04
	Control	14 (37.8)	23 (62.2)	
≥ 45░yr	PCOS	12 (44.4)	15 (55.6)	0.61
	Control	16 (51.6)	15 (48.4)	

Data expressed as n (%); Chi-squared test

**Table III:** Multivariate logistic regression analysis by considering the breast density (low/high) as the dependent variable

Covariates	OR	95% CI	P-value
BMI	0.87	0.79–0.95	0.002
Vitamin D intake	0.35	0.16–0.81	0.01
The family history of breast surgery	2.54	0.96–6.67	0.06

Variables age, group (PCOS/control), BMI, WHR, vitamin D intake, physical activity, and positive family history of breast surgery were entered to the model

OR: Odds ratio; CI: Confidence interval; BMI: Body mass index; PCOS: Polycystic ovarian syndrome

**Table IV:** Comparison of variables two groups of the low- and high-density of breast

Variables	Low density (n░=░68)	High density (n░=░68)	P-value
Age^*^			
< 45░yr ≥ 45░yr	40 (58.8) 28 (41.2)	38 (55.9) 30 (44.1)	0.86
BMI^*^			
18.5–24.99 25–29.99 ≥ 30	15 (22.1) 23 (33.8) 30 (44.1)	22 (32.8) 32 (47.8) 13 (19.4)	0.009
Age of Menarche^**^	13.56░±░2.49	13.78░±░1.75	0.55
Age at first delivery^**^	23.26░±░6.67	22.23░±░5.27	0.39
WHR^**^	0.87░±░0.07	0.84░±░0.09	0.07
Regular menstruation^*^	35 (51.5)	33 (48.5)	0.86
Vitamin D consumption^*^	14 (20.6)	28 (41.2)	0.02
Calcium consumption^*^	56 (82.4)	59 (86.8)	0.64
The positive family history of breast surgery^*^	8 (11.8)	19 (27.9)	0.03
Daily sun exposure more than 30░min^*^	10 (14.7)	9 (13.2)	1
Exposure to cigarette smoke^*^	15 (22.1)	15 (22.1)	1
Physical activity^*^	45 (66.2)	37 (54.4)	0.22
History of breast feeding^*^	50 (73.5)	56 (82.4)	0.30
History of abortion^*^	21 (30.9)	17 (25)	0.57

* Data expressed as number (%);

** Data expressed as mean░±░SD

P-value refers to *t*-test in comparison of continues variables and Chi-square test was conducted between categorical variables

BMI: Body mass index; WHR: Waist-hip ratio

## 4 Discussion

In the present study, we evaluated the breast density as a well-known risk factor of breast cancer in PCOS patients compared with the normal group in Iranian women. We reported the statistically significant difference in the breast density between PCOS and control group (Table I). As we mentioned in Table I, 23.5% of PCOS patients belong to the lower category of ACR breast density in comparison with 5.9% in control group, and ACR category of 3 was higher in control group in comparison with the case group (44.1% vs 30.9%). Since the previous epidemiological study reported high breast density is a risk factor of breast cancer, and our finding didn't confirm the high breast density in PCOS patients, our data suggest that PCOS patients are not at an increased risk of breast cancer due to breast density. Therefore, it seems that other mechanisms except breast density should be investigated in the PCOS population in order to detect the relationship between the breast cancer and PCOS.

Our results support several investigations that show women with PCOS are not at increased risk of breast cancer (10–12, 14). Similar to the present study, the association between PCOS and breast cancer risk in a meta-analysis was estimated at 0.87 (95% CI, 0.44–1.31). Although the finding of the study is not statistically significant, they created a hypothesis about the protective effect of PCOS in breast cancer risk (21). The anovulatory and irregular menstrual cycle is common in PCOS patients. Our finding is consistent with the hypothesis which reported that the reduced exposure to ovulatory menstrual cycles period is a protective effect against breast cancer (22). Two studies displayed results in contradiction with the present study. They reported the possible association between PCOS and breast cancer (13, 23). Kim *et al.* reported that PCOS and PCOS-related symptoms may play a role in the development of premenopausal breast cancer (13).

In this study, we found that the cigarette smoke exposure in PCOS patients was higher than in the control group with a borderline p-value (0.06), and the breast density in PCOS group was lower than the control group. This result supported previous evidence about the relationship between smoking and breast density that reported the lower measure of breast density in a current smoker than non-smokers (24–26). Since the exposure to estrogen has been associated with breast density positively, these protective effects of smoking might be due to the anti-estrogenic effect of cigarette smoking (27). Therefore, higher exposure to cigarette smoke in PCOS patients may cause decreased breast density in this group. As we found in multivariate analysis, BMI is inversely associated with breast density and it was confirmed by another study (5). Because one investigation has shown metabolic syndrome and its components such as insulin resistance were related to the dense breast (23) and PCOS disease is associated with a variety of clinical and laboratory findings such as hyperandrogenism and insulin resistance, we expected to observe the dense breast in PCOS patients. However, the present results have not confirmed this opinion. Because we did not have access to the hormonal profiles such as insulin resistance in PCOS patients, we are not able to investigate this issue.

Considering our result, vitamin D intake has a negative relation with breast density (OR░=░0.35, 95% CI: 0.16–0.81). This issue was confirmed by the other investigations which suggested that the dietary vitamin D could reduce the risk of breast cancer (28, 29). Ziv *et al.* revealed that women with higher breast density were more likely to have first-degree relatives with breast cancer (30), and this study showed that women who had positive family history of any breast surgery are at increased risk of high-density breast with OR equal to 2.54 (95% CI: 0.96–6.67) and a borderline p-value (0.06). Finally, PCOS patients of the present study had more physical activity compared with the control group. Epidemiological studies manifested that the increased physical activity declines the mammographic dense area (31, 32). Further studies are needed to confirm this association in premenopausal women.

## 5 Conclusion

The low breast density in PCOS samples of this study may relate to hormonal disorders and anovulation cycles, cigarette smoke, BMI, vitamin D intake, and physical activity. However, the final analysis did not manifest the association between groups (PCOS/control) and breast density considering other independent variables.

Our study displayed various advantages. Firstly, it was the first evaluation of breast density in PCOS patients in Iranian women population, and based on our knowledge, we didn't find any other studies on this topic. Another advantage of the present study was that all mammographic density assessment was performed by one expert radiologist. This study had some limitations. The first limitation was about the power of our study and we couldn't reach our estimation for sample size calculation (25% difference between high or low breast densities between two groups). Since the age of routine mammography in Iran is 40░yr, only women who were older than 40 entered the study due to budget and time limitations. Another limitation was the lack of information about hormonal profiles of all participants. It would be better to conduct further studies by considering the complete hormonal profiles of the sample population such as serum follicle-stimulating hormone (FSH), luteinizing hormone (LH), progesterone, estradiol, testosterone, and dehydroepiandrosterone sulfate. This study supported the role of vitamin D in order to decrease the breast density as a risk factor of breast cancer. Meanwhile, screening mammography should be recommended in women who had a family history of breast disease. It seems there are not sufficient data about the risk of breast cancer in PCOS patients and more investigations in different ages considering all confounding variables are necessary.

## Conflict of Interest

The authors declare that there is no conflict of interest.
